# Validation of a model for optimal birth weight: a prospective study using serial ultrasounds

**DOI:** 10.1186/1471-2431-12-73

**Published:** 2012-06-15

**Authors:** Gavin Pereira, Eve Blair, David Lawrence

**Affiliations:** 1 Telethon Institute for Child Health Research, Centre for Child Health Research, University of Western Australia, 100 Roberts Road, Subiaco, WA, 6008, Australia; 2 Yale School of Public Health, Center for Perinatal, Pediatric and Environmental Epidemiology, Yale University, New Haven, CT, 06510, USA

**Keywords:** Fetal growth, Preterm delivery, Proportion of optimal birth weight

## Abstract

**Background:**

The aim of this study was to validate a model for optimal birth weight derived from neonatal records, and to test the assumption that preterm births may be considered optimally grown if they are not exposed to common factors that perturb fetal growth.

**Methods:**

Weights of fetuses were estimated from serial biometric ultrasound scans (N = 2,848) and combined with neonatal weights for a prospective pregnancy cohort (N = 691). Non-Caucasians, fetuses subsequently born preterm and those with diagnosed or suspected determinants of aberrant growth were excluded leaving fetuses assumed to have experienced normal growth. A generalised linear longitudinal growth model for optimal weight was derived, including terms for gestational duration, infant sex, maternal height and birth order. This model was compared to a published model derived solely from birth weights.

**Results:**

Prior to 30 weeks gestation, the published model yielded systematically lower weights than the model derived from both fetal weight and neonatal weight. From 30 weeks gestation the two models were indistinguishable.

**Conclusion:**

The model for optimal birth weight was valid for births that have attained at least 30 weeks gestation. The model derived from both fetal and neonatal weights is recommended prior to this gestation.

## Background

Standards for fetal weight are typically derived from birth weights of neonates born at different gestational ages [1,2]. However, births at early gestations are frequently affected by pathologies that restrict growth [3-5]. Therefore standards of growth derived from birth weights will tend to under-estimate the weight of unborn fetuses of the same gestation, thereby under-estimating the degree of growth restriction in infants born preterm [6]. Such situations may lead to inappropriate counselling and planning for preterm delivery [6]. This dilemma is insurmountable by studies considering only birth weight since if preterm births were excluded no estimate of growth would be available at early gestational ages.

Optimal weight may be interpreted as a result of growth achieved in the absence of any factors that pathologically affect growth. We have previously reported a method of assessing the appropriateness of fetal growth using the proportion of optimal birth weight [7]. With that approach the measure of growth was the ratio of the observed birth weight to the estimated optimal birth weight given the neonate’s non-pathologic determinants of growth. In that study ‘optimal’ weight was defined as the weight achieved by neonatal survivors not exposed to any of the exposures associated with intrauterine growth anomaly commonly occurring in our population; namely: maternal smoking, vascular disease, diabetes (pre-existing or gestational), TORCH infections (toxoplasmosis, rubella, CMV, herpes) in pregnancy, multiple pregnancy and birth defects in the fetus. Gestation of delivery was not a criterion for optimal growth. As anticipated, a far greater proportion of preterm births were excluded by these criteria than were term births, but we had no compelling reason for excluding other preterm births on the grounds of growth anomaly, despite their preterm birth suggesting experience of suboptimal exposures. However the assumption that neonatally surviving preterm births not exposed to common causes of growth anomaly are optimally grown was untested.

The aim of this study was to validate a model for optimal birth weight derived from neonatal records, and to query the assumption that preterm births may be considered optimally grown if they are not exposed to common factors that perturb fetal growth.

## Method

Our analytic approach is summarised by the following steps:

i. We selected a sample of optimally grown term births from a randomised controlled trial of serial biometric ultrasound scans in pregnancy. Weight was estimated from biometric measurements made during each ultrasound scan during pregnancy and obtained at the time of birth.

ii. A model for optimal weight was derived at each of these measurement occasions using the fetal and neonatal weights as the response variable and non-pathologic determinants of growth as explanatory variables. OW_US_ refers to optimal weights derived with this model.

iii. Optimal weight was then derived at each of these measurement occasions using the published model derived using only birth weights from an independent population, OW_BW_.

iv. The difference in optimal weight estimated by the two models was calculated at each week of gestation.

### Sample selection

**Figure 1:**
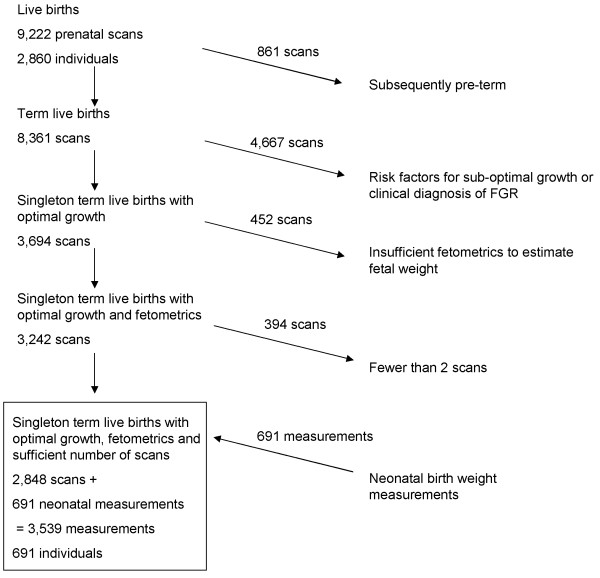
Flow diagram showing exclusions and final sample.

A total of 9,222 serial ultrasound scans were obtained between 1989 and 1991 in Perth Western Australia for 2,860 pregnancies that resulted in a live birth (Figure 1). The cohort were recruited through the Raine Study, which was a randomised trial initially designed to study fetal outcome in relation to the influence of multiple ultrasound scans (scheduled for weeks 18, 24, 28, 34 and 38 weeks gestation). We excluded scans of fetuses subsequently born preterm.

We then restricted the population to Caucasians and excluded scans of pregnancies complicated by maternal factors associated with growth anomaly (Table 1): maternal smoking, maternal vascular disease (essential hypertension only, gestational hypertension only, gestational hypertension with proteinuria (preeclampsia), essential hypertension with proteinuria), pre-existing or gestational diabetes, multiple pregnancy. Next, scans for a fetus subsequently diagnosed with fetal growth restriction (FGR) under clinical assessment were excluded. Clinical assessment for FGR involves physical examination of the neonate postpartum.

**Table 1:** Number of scans and individuals by risk factor for suboptimal growth and clinical diagnoses of FGR among singleton liveborn term neonates

	**Scans**		**Individuals**	
	**N**	**%**	**N**	**%**
Singleton liveborn neonates	8,361	100	2496	100
Non-Caucasian	810	10	242	10
Maternal smoking	2,158	26	658	26
Maternal vascular disease*	2,140	26	602	24
Congenital anomaly	24	0	18	1
Maternal pre-existing or gestational diabetes	338	4	80	3
Clinically assessed FGR	224	3	66	3
Any of the above risk factors	4,667	56	1365	55

*Essential hypertension only, gestational hypertension only, gestational hypertension and proteinuria (preeclampsia), essential hypertension and proteinuria.

In order to allow for curvature in the growth curves we restricted analysis to individuals who had at least 2 scans i.e. at least 3 weight measurements after including the neonatal record of birth weight. Remaining for analyses were 2,848 serial ultrasound scans for 691 individuals. Neonatal records of birth weights and associated gestational ages (N = 691) were appended to the ultrasounds (N = 2,848), resulting in 3,539 measurements (Table 2).

**Table 2:** Gestational age distribution for (i) the final study cohort, and (ii) the previous study cohort used to derive a model for optimal weight using only neonatal measurements

**Gestation**	**Study cohort**		**Previous study cohort**	
	N*	**%**	N**	**%**
14–17 weeks	147	4	0	0
18–22 weeks	578	16	0	0
23–27 weeks	561	16	76	0
28–32 weeks	550	16	258	0
33–39 weeks	1,251	35	35,575	57
40–42 weeks	452	13	26,837	43
Total	3,539		62,746	

* Scans and neonatal measurements.

** Neonatal measurements.

### Calculation of fetal weight from ultrasound scans

Abdominal circumference (AC), femur length (FL), head circumference (HC) and biparietal diameter (BPD) measurements were obtained from the ultrasounds. Hadlock’s formula was applied to estimate fetal weight using AC, FL, HC and BPD measurements taken at or after 27 weeks gestation [8]. In a comparison of 12 equations for fetal weight, Hadlock’s 4-variable formula performed well in terms of error bias and error precision for subsequent births over 1000 g [9]. Scott’s formula was applied to estimate fetal weight using AC, FL and HC measurements taken prior to 27 weeks gestation (189 days) as it proved the most accurate in a comparison with 10 other equations at early gestations [10].

### Estimation of optimal weight derived from both ultrasound and neonatal measurements

A predictive model for the expected fetal weight (OW_US_) derived from both the fetal ultrasound scans and neonatal measurements was determined using gestational age, maternal height, birth order and infant sex as predictors. All first order interactions between the predictor variables were considered along with polynomial forms of gestational age. Box-Cox transformations were applied to improve the fit of the model [11]. Despite transformations, the error variance of the growth data increased with the mean. Therefore, a generalized linear model was applied using a gamma distributed response variable and a log link function, which subsequently eliminated heteroskedasticity. As there were repeated ultrasound measures for each fetus, the covariance among the errors for the same individual were assumed to have a power covariance function of the form:

(1) $$Cov(\epsilon_{t,j},\epsilon_{t*,j})=\sigma_{\epsilon }^{2}\rho^{d_{t,t*}}$$

where *ρ* is the correlation between fetal weight measurements taken a day apart and *d*_t, t*_ is the interval of time between time t and time t*. Therefore, measurements taken closer in time were assumed to be more similar than those further apart. The analysis was conducted using the GLIMMIX procedure of SAS version 9.1 [12].

### Estimation of optimal weight derived from neonatal measurements only

The optimal weight (OW_BW_) was calculated for each record using a previously published model that relied on birth weight measurements [7]:

(2) $$\text{O}{\text{W}}_{\mathrm{BW}}={\left[\begin{array}{l} -1 4.0 8-1 4 1 4{\left(\frac{\text{GA}}{100}\right)}^{3}-2 7 8 2{\left(\frac{\text{GA}}{100}\right)}^{3}\text{ln}\left(\frac{\text{GA}}{100}\right)+1.1 8 5\text{M a l e}+0.1 0 7 7(\text{H e i g h t}-1 6 2)\\ +1.0 2 8\text{S e c o n d}+1.3 1 8\text{T h i r d}+1.5 7 1\text{M o r e}+0.0 0 6 6 7(\text{GA}-40)(\text{Height}-162) \end{array}\right]}^{2}$$

where GA refers to the gestational age at which the measurement was taken, Male is an indicator variable for male sex, Second refers to the second birth (para 1), Third refers to the third birth (para 2), and More refers to the fourth birth or more. Height is maternal height measured during pregnancy.

In that study, similar exclusion criteria were applied: multiple birth (3%), maternal smoking during pregnancy (27%), maternal vascular disease (7%), congenital anomaly (6%) and maternal pre-existing or gestational diabetes (4%) [7]. The prevalence of maternal vascular disease was higher in this study due to the inclusion of essential hypertension (Table 1).

### Comparison between the OW_US_ and OW_BW_

The proportional deviation of OW_BW_ from OW_US_ was calculated at each gestational age as:

(3) $$\text{Proportional}\;\text{Deviation}=\frac{OW_{\mathrm{BW}}-OW_{\mathrm{US}}}{OW_{\mathrm{US}}}$$

The formula for OW_BW_ was considered an appropriate representation of OW_US_ at gestational ages where the 95% bootstrap confidence interval [13] for the proportional deviation intersected zero.

### Approvals

Ethics approval was obtained by the Department of Health Western Australia, Human Research Ethics Committee.

## Results

### Sample population and ultrasound scan characteristics

There was an approximately equal ratio of male to female births (51:49) (Table 3). Mothers were most likely to be in the 20–29 year (30%) and 30–34 year (29%) age groups. Approximately 72% of the women had at least 4 scans. The 14–17 week and 40–42 week gestational periods were represented by only 147 (5%) and 21 (1%) scans respectively.

**Table 3:** Study characteristics

**Characteristic**	**N**	**%**
All births	691	100
All scans	2,848	100
Infant sex		
Male	354	51
Female	337	49
Maternal age (years)		
Less than 20	58	8
20–24	132	19
25–29	210	30
30–34	201	29
35 or over	90	13
Birth order		
First birth	308	45
Second birth	215	31
Third birth	108	16
Fourth birth or more	60	9
Scans per gestation period		
14–17 weeks	147	5
18–22 weeks	578	20
23–27 weeks	561	20
28–32 weeks	550	19
33–39 weeks	991	34
40–42 weeks	21	1
Scans per individual		
2–3 scans	190	28
4–5 scans	459	66
6–8 scans	42	6

### Model for expected fetal weight from ultrasound scans

The Box-Cox procedure suggested the square root as the optimal transformation. The Pearson correlation between the observed and predicted fetal weights was 0.97. The square of this correlation, a pseudo R-squared, indicated that the model explained 95% of the variation in fetal weights. The autocorrelation in the residuals between consecutive days was low but statistically significant, and therefore systematic and non-ignorable (*ρ* = 0.007191, SD = 0.001657).

Parameter estimates are shown in Table 4 and the model for OW_US_ (grams) expressed as:

(4) $$\text{EF}W_{\mathrm{US}}=\text{exp}2\left[\begin{array}{l} \beta_{0}+\beta_{1}\left(\frac{\text{Time}}{300}\right)+\beta_{2}{\left(\frac{\text{Time}}{300}\right)}^{2}+\beta_{3}{\left(\frac{\text{Time}}{300}\right)}^{3}+\beta_{4}{\left(\frac{\text{Time}}{300}\right)}^{4}\\ +\beta_{5}\text{Male}+\beta_{6}\text{Second}+\beta_{7}\text{Third}+\beta_{8}\text{More}+\beta_{9}(\text{Height}-162)\\ +\beta_{10}\text{Time}(\text{Height}-162) \end{array}\right]$$

where Time is the time of gestation represented in days since the last menstrual period.

**Table 4:** Parameter estimates for the square root of optimal fetal weight (grams)

				**95% CI**		
**Parameter**	**Variable**	**Estimate**	**SE**	**Lower**	**Upper**	***p*-value**
*β*_0_	Intercept	−4.311	0.2097	−4.7223	−3.8997	<.0001
*β*_1_	$\frac{\text{Time}}{300}$	35.4907	1.3364	32.8702	38.1111	<.0001
*β*_2_	${\left(\frac{\text{Time}}{300}\right)}^{2}$	−66.8095	3.0939	−72.8763	−60.7428	<.0001
*β*_3_	${\left(\frac{\text{Time}}{300}\right)}^{3}$	61.7716	3.0934	55.7058	67.8373	<.0001
*β*_4_	${\left(\frac{\text{Time}}{300}\right)}^{4}$	−22.0933	1.1304	−24.3099	−19.8767	<.0001
*β*_5_	Male	0.0112	0.003276	0.004766	0.01763	0.0007
*β*_6_	Second birth	0.001354	0.003832	−0.00617	0.008876	0.7239
*β*_7_	Third birth	0.01575	0.004821	0.006291	0.02522	0.0011
*β*_8_	Fourth birth or more	0.02281	0.006053	0.01093	0.03469	0.0002
*β*_9_	Maternal height -162	−0.00155	0.000729	−0.00298	−0.00012	0.0336
*β*_10_	Time (Maternal height – 162)	0.003979	0.001003	0.002012	0.005945	<.0001

### Comparison between the OW_US_ and OW_BW_

**Figure 2:**
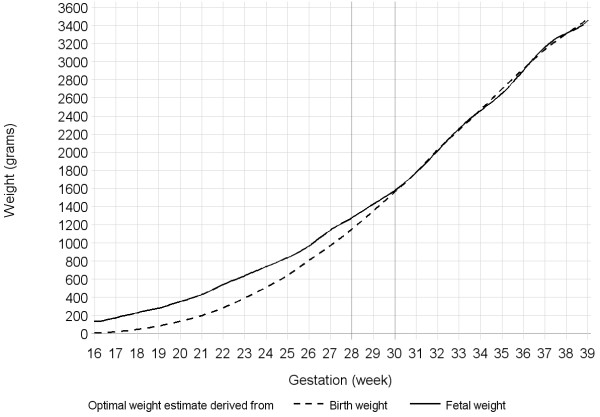
**Curves for the mean OW**_**BW**_**and OW**_**US**_**by gestational age.**

**Figure 3:**
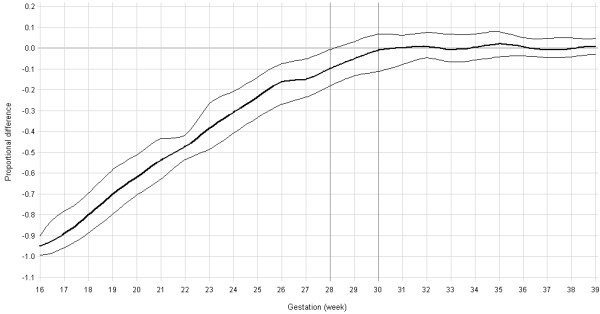
**Proportional deviation of OW**_**BW**_**from OW**_**US**_**by gestational age with 95% Bootstrap confidence interval bounds.**

The curves for mean OW_BW_, OW_US_, and proportional deviation of OW_BW_ from OW_US_ were calculated from 16 to 39 weeks gestation because few ultrasound scans were conducted outside this interval (Table 3). The curves for mean OW_BW_ and OW_US_ differed before 28–30 weeks when OW_BW_ approached zero (Figure 2). The OW_BW_ was systematically lower than OW_US_ before 30 weeks gestation and the difference was statistically significant prior to 28 completed weeks of gestation (Figure 3). The difference between OW_BW_ and OW_US_ increased by as much as 10% per earlier week of gestation.

### Sensitivity of the results to the choice of model used to estimate fetal weight

Hadlock’s formula was applied to estimate fetal weight from ultrasound scans at or after 27 weeks gestation [8] and Scott’s formula was applied at earlier gestations [10]. We repeated the study using Hadlock’s formula instead of Scott’s formula to estimate fetal weight from scans taken before 27 weeks gestation. The mean difference between estimated fetal weight based on Scott’s model and Hadlock’s model (*Scott* - *Hadlock*) was non-zero (*p* < 0.0001) at 10 g (SD = 26 g) using fetometric ultrasound measurements taken before 27 weeks gestation. The mean difference between estimated fetal weight based on Scott’s model and the previously published model (*Scott* – OW_BW_) was also non-zero (*p* < 0.0001) at 197 g (SD = 68 g) using fetometric ultrasound measurements taken before 27 weeks gestation.

## Discussion

Our study made use of a large prospectively collected sample of serial ultrasounds to derive an estimate of the optimal fetal weight and thereby also a method to ascertain the adequacy of fetal growth. We adopted a multi-faceted approach: weight was derived from ultrasound scans taken at multiple occasions during pregnancy and supplemented with weight measured at the time of birth, rather than sole reliance on birth weights and we excluded fetuses subsequently (i) born preterm, (ii) that died before 28 days of life, or (iii) that experienced pathologies affecting growth. We found that estimates of optimal weight based on a population of birth weights also subject to exclusions (ii) and (iii) but not (i) were systematically lower than fetal weight estimated using biometric ultrasounds prior to 30 weeks gestation.

It is plausible that the difference between OW_US_ and OW_BW_ (Figure 2, Figure 3) suggests an increasing proportion of unusual causes of growth restriction with decreasing gestation of delivery before 28 to 30 weeks gestation, but very few such causes of growth restriction for births after 30 weeks. We re-examined those in the original cohort used to derive OW_BW_ that were born at or before 28 weeks gestation (N = 101). Among this group, the recorded antepartum factors that might have contributed to growth restriction but were not excluded as common causes of growth restriction were: threatened abortion (antepartum haemorrhage before 30 weeks gestation), N = 13; urinary tract infection, N = 2; antepartum haemorrhage (not attributed to placenta previa or abruption), N = 39; asthma, N = 12; genital tract infection, N = 9; vaginitis, N = 4; significant psychological morbidity, N = 11; anaemia, N = 5; neoplasms, N = 7. Approximately 63% of this cohort experienced at least one of these factors. Among all term neonates in Western Australia, the prevalence of asthma was 9.7% and neoplasms (cervical cancer and cervical dysplasia) was 0.02%, which were both lower than the prevalence in this cohort. Further studies are required to confirm whether maternal asthma, cervical cancers and depression are disproportionately stronger risk factors for growth restriction at such early gestations in other populations. It remains to be demonstrated that preterm births, particularly very preterm births, can be considered optimally grown if they are not exposed to common factors that perturb fetal growth.

Although it is plausible that there is an increasing proportion of unusual causes of growth restriction with decreasing gestation of delivery before 28 to 30 weeks gestation, there are other explanations for the findings. Radiographers may have systematically over or under-estimated fetal biometric measurements. However, the measurements were taken by a limited number of experienced radiographers at a tertiary obstetric hospital. While both Hadlock’s and Scott’s models were selected because they performed favourably compared to alternative formulae, it is possible that both of these methods used to estimate fetal weight from ultrasound measurements systematically over-estimated true fetal weight in our study. Few ultrasound scans were taken within a week of birth among early *low-risk* preterm births before 28 weeks gestation. This meant that a formal validation of the model used to estimate fetal weight from ultrasound measurements could not be conducted. However, we confirmed that results were not sensitive to the choice of Hadlock’s versus Scott’s method to estimate fetal weight from ultrasound measurements. Before 27 weeks gestation, Scott’s method and Hadlock’s method to estimate fetal weight from ultrasound measurements differed by only 10 g, whereas the difference between estimates obtained from Scott’s method and the model for optimal weight derived from birth weight measurements was almost 200 g.

A further alternative explanation for the findings of this study is that the model developed using a combination of fetal weights derived from ultrasound scans and birth weights fits the data better at earlier gestations than the published model derived from birth weights due to the small number of births at early gestations. The model for OW_BW_ was developed predominantly on a sample of births from 33 weeks gestation whereas the model for OW_US_ was based on measurements from ultrasounds that start typically from much earlier gestations (Table 2). Therefore, the model for OW_US_ is recommended for the estimation of fetal weight instead of OW_BW_, particularly before 30 weeks gestation.

Our results support the overall findings of Salomon et al (2007) who reported that the median of the fetal weight distribution provided an upper bound for the median of the birth weight distribution between 25 and 35 weeks of gestation [6]. However, their inclusion of individuals subsequently born pre-term, those exposed to maternal smoking during pregnancy, and those with known growth restricting pathologies would have deflated the true discrepancy. Not accounting for known non-pathological determinants of growth such as birth order and maternal stature would have introduced further error. Moreover, the proportional difference between the two approaches may be of greater interest than the absolute difference as it is a measure of difference relative to the fetal size. Our results indicate that the proportional deviation of OW_BW_ from OW_US_ was statistically significant prior to 28 weeks gestation, after accounting for individualised growth potential and excluding those with diagnosed growth restriction or known growth restricting pathologies. From 30 weeks of completed gestation the estimates of fetal weight based on a population of birth weights not exposed to common causes of growth anomaly yielded similar estimates of optimal weight.

The methodology that we applied differs from those suggested by others in that centiles and z-scores were not produced [14]. However, the aim of our study was to compute and compare the mean optimal fetal weight derived from both ultrasounds and neonatal measurements to that derived from only birth weights, rather than produce reference charts and compare the entire distributions. Nonetheless, we responded to the recommendations of Altman et al (1994) as we fully accounted for the non-constant variance of the residuals with increasing gestational age [15]. Albeit small in magnitude, the temporal autocorrelation among the fetal weights was statistically significant after accounting for gestational age, birth order, infant sex and maternal height. Therefore, past studies that ignored the temporal autocorrelation violated this requirement for regression. A limitation of our approach is the increased complexity of modelling the error variance. A further limitation of our study was that the exclusion criteria restricted the sample size from 9,222 scans to 2,848 scans. However, this meant that the expected optimal fetal weight estimates were less likely to be influenced by individuals with pathologically affected growth. The prospective design also allowed the retention of multiple scans per individual despite these exclusions.

## Conclusion

It remains to be demonstrated that preterm births can be considered optimally grown if they are not exposed to common factors that perturb fetal growth. The model derived from both fetal and neonatal weights in this study is recommended before 30 weeks of gestation. From 30 weeks gestation the two models were indistinguishable.

## Competing interests

The authors declare that they have no competing interests.

## Authors’ contributions

GP participated in the design of the study, performed the statistical analysis, and wrote the initial manuscript and later revisions. EB conceived the study, participated in its design and helped to draft and revise the manuscript. DL participated in the design of the study and helped to revise the manuscript. All authors read and approved the final manuscript.
